# Genetic diversity through social heterosis can increase virulence in RNA viral infections and cancer progression

**DOI:** 10.1098/rsos.202219

**Published:** 2021-05-05

**Authors:** Saba Ebrahimi, Peter Nonacs

**Affiliations:** Department of Ecology and Evolutionary Biology, University of California, 621 Young Drive South, Los Angeles, CA 90024, USA

**Keywords:** genetic diversity, social heterosis, cancer, metastasis, virus, virulence

## Abstract

In viral infections and cancer tumours, negative health outcomes often correlate with increasing genetic diversity. Possible evolutionary processes for such relationships include mutant lineages escaping host control or diversity, *per se*, creating too many immune system targets. Another possibility is social heterosis where mutations and replicative errors create clonal lineages varying in intrinsic capability for successful dispersal; improved environmental buffering; resource extraction or effective defence against immune systems. Rather than these capabilities existing in one genome, social heterosis proposes complementary synergies occur across lineages in close proximity. Diverse groups overcome host defences as interacting ‘social genomes’ with group genetic tool kits exceeding limited individual plasticity. To assess the possibility of social heterosis in viral infections and cancer progression, we conducted extensive literature searches for examples consistent with general and specific predictions from the social heterosis hypothesis. Numerous studies found supportive patterns in cancers across multiple tissues and in several families of RNA viruses. In viruses, social heterosis mechanisms probably result from long coevolutionary histories of competition between pathogen and host. Conversely, in cancers, social heterosis is a by-product of recent mutations. Investigating how social genomes arise and function in viral quasi-species swarms and cancer tumours may lead to new therapeutic approaches.

## 1. Introduction

Disease is a competitive game between a host and its invading pathogens, or similarly, between normal body cells and mutated cancer cells. To the degree that one side wins, the other loses. Typically, there is only one ‘host’ side, but the ‘disease’ side can have multiple players. Different pathogens can co-infect the same host, and cancer cells mutate to form genetically differentiated cell lineages. Although these players do share a common goal—to overcome the host's defences—they may also find themselves in a version of the ‘Tragedy of the Commons' game [1–3]. The more resources that one pathogen or one cancer cell lineage draws from its host, the less is left over for any others. Genotypes that replicate more rapidly are at an evolutionarily selective advantage over those that are more restrained. If this is a zero-sum competition, it can lead to the evolution of particularly virulent genomes.

An alternative evolutionary possibility for the disease players is one of social heterosis [4–6]. The essence of social heterosis is that genetically diverse groups can be more successful and productive than genetically homogeneous ones. This can result in several ways. First, diverse groups can reduce within-group competition when individuals no longer completely overlap in their resource needs. Second, diversity can increase homeostasis through better environmental buffering or defence against predators or disease. Third, diverse groups can have a larger genetic ‘tool kit’ and, therefore, an enhanced skill set for productive activity. Social heterosis can produce group phenotypes that are more expansive and multifaceted than any single genome can be. To the degree that such groups gain any or all of these benefits, we can consider their entire genetic complement to be a ‘social genome’ [6].

Genetic diversity can also produce enhanced fitness outcomes that are not due to social heterosis. For example, multiple pathogen clones or cancer cell lineages can interact to produce a ‘rescue’ effect. Deleteriously mutated pathogens or cancer cells that cannot perform some critical function can still survive and replicate if the presence of others provides the missing function. This, however, would not be social heterosis. For social heterosis, genetically diverse assemblages must do more than survive—they must be more productive than any single, isolated genotype. There must be true ‘complementarity’ that increases group replicative success to be strong evidence for social heterosis.

**Figure 1.**
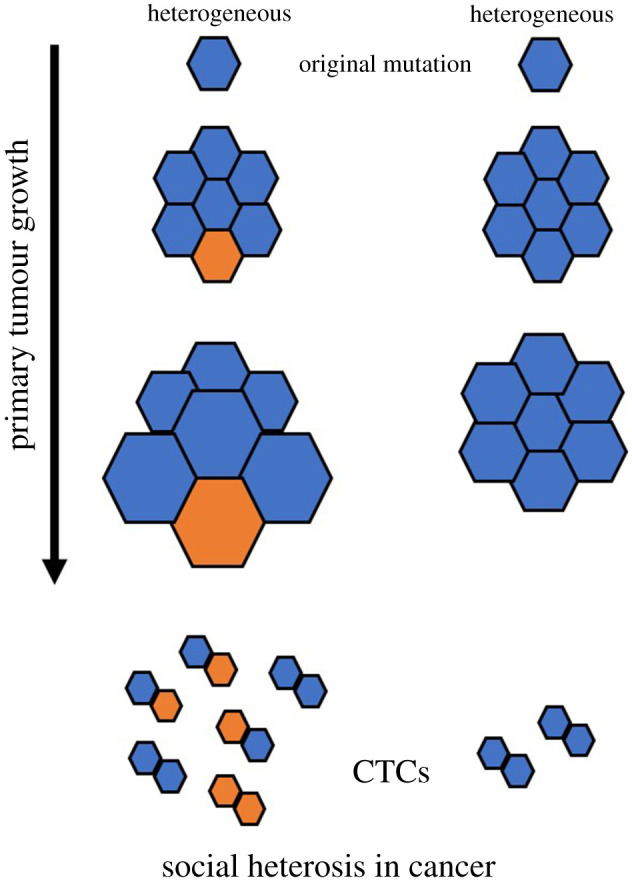
Social heterosis in cancer tumours and metastasis. Genetically diverse ‘neighbourhoods’ within a tumour can replicate more rapidly due to mutated cell lineages having a greater tool kit in terms of extracting resources from the host or defending against immune system responses. Although each lineage may only have a specific genetic advantage, the benefits produced by those enhanced capabilities are shared by all cells in close proximity. Thus, as the tumour grows, genetic diversity is maintained and entire neighbourhoods can function as complementary social genomes. Metastatic ability and success can also be enhanced in polyclonal circulating tumour cells (CTCs) relative to monoclonal CTCs.

**Figure 2.**
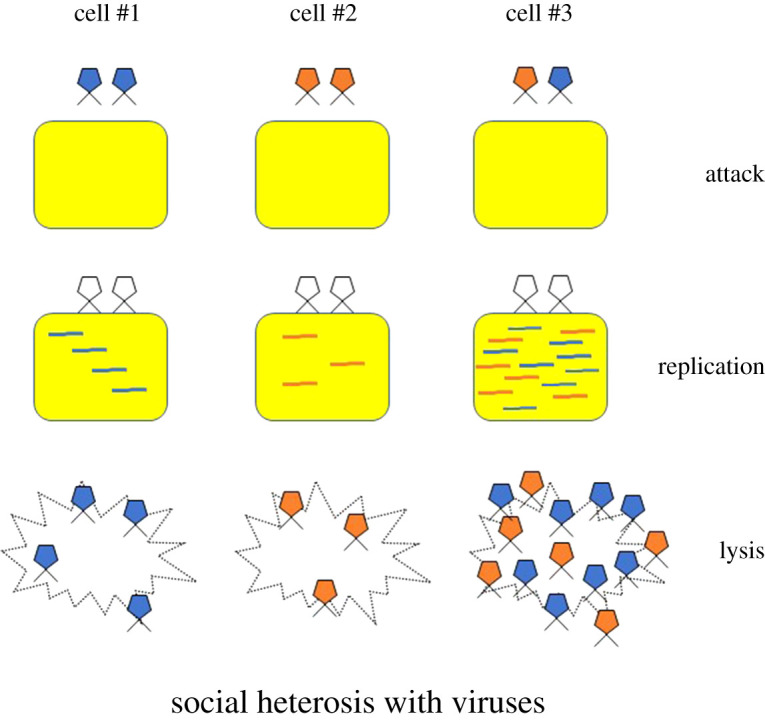
Social heterosis in viruses. When cells are co-infected by genetically different clones, the DNA or RNA genomes can interact as a polyploid social genome. With this greater amount of genetic information, the viral infection may enhance replication for all infecting genotypes and, therefore, release more virions upon cell lysis. Social heterosis can select for and maintain genetic diversity even under conditions where not all genotypes are replicating at the same rate within cells [4,5]. If heterogeneous co-infections are overall more productive than homogeneous ones, group-level selection can overcome within-cell individual-level selection for replicative success. Note that in some viruses, co-infection can also result from more than one genome being packaged in protein capsids.

In higher organisms, social heterosis can reflect both morphological and behavioural diversity, the latter of which can be considered in the context of overtly cooperative decisions [7]. This is not so in the case when applying social heterosis to cancer or viral infections. Here, the more applicable construct is one of an expanded tool kit. Genetically different clones do not decide in any formative way to cooperate. Instead, social heterosis in a cancer tumour would be a by-product of genetically differentiated, but physiologically complementary, cells that reside in the same local neighbourhood (figure 1). Some cells might create a defence against being attacked by the immune system, while others increase the rate at which resources are extracted from adjacent non-cancerous tissue [8]. Similarly, if viral clones that co-infect the same cell are complementary in using the cell's resources, replication rates of all genotypes could be enhanced due to their synergistic skill sets (figure 2).

Although heterogeneity can make a complementary social genome, not all genotypes are likely to have equal replicative rates. Therefore, a social genome would always be at risk of collapsing from within-group selection for the best replicator to increase to fixation, even at a cost of eliminating all the benefits of diversity. Only if the population has across-group variation in genotypes and a mixing phase in propagule dispersal (i.e. the population exists as structured deme), can group-level selection counteract individual within-group selection [4,9]. This seriously constrains the possibility of evolution through social heterosis. Group members reproduce, groups do not. Genotypes, not social genomes, reproduce. A group of cells comprising a cancer tumour can metastasize through sloughing off circulating tumour cells (CTCs). Any given CTC, however, may not incorporate the complementary subsets of genotypes that comprise a social genome. Similarly, co-infecting and complementary viruses may replicate effectively when together in the same cell, but whether or not their descendent virions find themselves similarly co-infecting other cells may be stochastic and determined by random chance. For viruses, when transmission involves getting from one host to another, the ensuing genetic bottlenecks may also severely limit the inheritance of intact social genomes.

In short, there are reasons why social heterosis might be an important feature in evolutionary medicine, but there are also reasons why it might not be. In a first attempt to test the relevance of social heterosis for disease outcomes, Nonacs & Kapheim [6] simulated the evolution of the HIV virus within hosts, with the possibility that mutations could produce complementary interactions across separate clone lines. The model outcomes accurately matched a number of features of HIV progression from a long and variable asymptomatic phase to properties of the viral population at the point of immune system collapse.

Applied more broadly, the social heterosis hypothesis leads to a number of testable predictions (table 1). Some are very general and could arise for a variety of reasons, such as pathogen populations or cancer tumours being polyclonal rather than monoclonal because of replicative dysfunction. Other predictions, however, are far more specific, such as heterogeneous groups exhibiting replicative complementation or evolutionary convergence of certain allele combinations across different hosts. We, therefore, expand on the previous application to HIV by searching the literature for other potential examples that are consistent with one or more of the social heterosis predictions (table 1). We consider two very different systems, viral infections, and cancer growth and spread. Each one has characteristics that seem favourable for creating for social heterosis: (i) the formation of localized groups of genetically variable virions or cell lineages; (ii) across-group variation in genetic diversity; and (iii) the potential that heterogeneity correlates with group-level virulence or tumour growth and metastasis. Finding multiple studies across different types of cancer and taxonomic groups of viruses that match predictions would suggest that social heterosis is indeed a relevant concept for evolutionary medicine to consider, especially in regard to future therapeutic practices.

**Table 1.** Social heterosis predictions.

**Overall patterns of genetic diversity consistent with social heterosis**
(1) Naturally occurring populations of pathogens are always diverse; genetic similarity or relatedness of pathogens negatively correlates with group-level success. [10].
(2) Diversity is always lowest when pathogens first infect hosts or at early stages of primary tumour growth and then significantly increases over time.
(3) Diverse pathogen or cancer cell populations are more often associated with increased replication and negative health outcomes than monoclonal populations.
(4) Transmission bottlenecks can disrupt social genomes, requiring *in situ* evolution through random mutations and stochastic co-occurrence of complementary genotypes. This could produce significant variance in pathogen virulence or metastatic tumour growth across hosts.
(5) Diversity varies across local, interacting neighbourhoods of pathogens or cells. This creates structured deme populations within hosts compatible with group-level selection.
(6) If host defences collapse or become ineffective, population-level diversity increases at a slower rate, stops increasing or declines in the presence of a complementary social genome.
**Interactions across genotypes or phenotypes consistent with social heterosis**.
(1) For pathogens, immediate virulence negatively correlates with the severity of a genetic bottleneck at transmission. For cancers, the likelihood of metastasis positively correlates with genetic heterogeneity of circulating tumour cells (CTCs).
(2) Complementary interactions occur between genetically different pathogens or cancer cells. Particularly compelling is where clones genetically differ at the same loci.
(3) No single clone strain, wild-type or consensus sequence will have equal or higher replicative success than at least one combination of genetically diverse clones.
(4) Genotypes at pathogen infection or early primary tumours may be rare or absent when infections become chronic or cancers become metastatic.
(5) Not all genotypes will have equal replicative success within a local group or cell aggregation. Genotype fitness may vary across tissues or in terms of effectively exploiting host resources or evading host immunological defences.
(6) Frequency-dependent selection across genotypes will be weak or non-existent. Absolute co-occurrence of complementary genotypes has a greater effect than relative frequencies.
(7) For some pathogens or cancers, social genomes require specific allelic combinations or mutation sequences, creating predictable evolutionary patterns in loci across hosts.

## 2. Methods

We initially screened the extant literature (to early 2020 in Web of Science, JStor and Google Scholar) on cancers and viruses for evidence of social heterosis by using the keywords ‘genetic diversity’, ‘cooperation’, ‘synergistic interactions', ‘mutualistic interactions’, ‘group diversity’, ‘quasispecies’ and ‘group selection’. After identifying relevant studies, we expanded our search to examine papers that cited these original papers. If the original papers reviewed the literature, we examined papers therein that seemed appropriate, but had not been identified in our initial search. The literature review is biased in that we primarily searched for evidence suggestive of social heterosis, rather than a comprehensive review of all the ways that cancers and viruses could potentially proliferate.

## 3. Results

The individual studies found in the literature search (tables 2 and 3) are listed with how consistent they are or are not with predictions from the social heterosis hypothesis (table 1).

**Table 2.** Summary of results in virus studies relative to evidence for social heterosis. The support is categorized relative to specific overall predictions (O) or about clonal interactions (I) from table 1.

genome: family	disease	results [study]	support for social heterosis (categories from table 1))
DNA	bacteriophage	Wild-type outcompetes mutant versions. [11]	No support; suggests individual-level selection across clones. (none)
DNA: Baculoviridae	attacks Lepidoptera	Co-infections increase lethality and produce greater viral yields compared with single-genotype infections. Usually, one genotype benefits more. [12]	Supports social heterosis, but group selection would be needed to prevent genotype fixation. (O3,I5)
DNA: Baculoviridae	attacks Lepidoptera	The infectiousness and pathogenicity of mixed genotype infections is greater than that of single-genotype infections. [13]	Diverse groups have higher productivity. (O3)
DNA: Herpesviridae	neonatal herpes	Intrahost genetic diversity is high throughout the viral genome. There is greater spread in culture, but it is unclear whether one or more variants are functionally significant. [14]	Diverse groups possibly have higher productivity. (O3)
DNA: Herpesviridae	mouse herpes	Multiple infection correlates with increased virus load and disease severity. Evidence for complementation not recombination. [15]	Evidence for complementation between genetically different clones. (I2)
DNA: Herpesviridae	herpes	A viral strain can be composed of a mixed population of viruses or undergo bottlenecks that lead to the creation of a homogeneous population. Variants can arise by genetic drift over passages in culture and can differ in phenotypes. [16]	Increasing diversity is a precondition for social heterosis. (O1)
DNA: Herpesviridae	herpes	Virus populations in chronic infections have high clonal diversity, possibly through recombination. [17]	Increasing diversity is a precondition for social heterosis. (O1)
DNA: Herpesviridae	chickenpox	Population diversity increases across *in vitro* passages in cell culture. [18]	Increasing diversity is a precondition for social heterosis. (O1)
DNA: Hepadnaviridae	hepatitis B	Initial rapid evolution and diversification at certain loci, that thereafter is much slower. [19]	Consistent with the prediction of how a social genome evolves. (O6,I7)
RNA: Flaviviridae	dengue	Defective stop codon transmitted and increasing in frequency: complementation suggested, but could be rescue. [20]	Diverse transmission only evidence for social heterosis. (O1)
RNA: Flaviviridae	dengue	Independent emergence and/or expansion of genome hotspot variants within each host. [21]	Convergent evolution during acute dengue across hosts suggests a similar social genome evolves. (I7)
RNA: Flaviviridae	dengue	Sequence diversity in the same range in patients with better or worse outcomes. [22]	Diversity by itself is not correlated to severity as predicted by social heterosis. (none)
RNA: Flaviviridae	hepatitis C	Consistent with sequential selective sweeps where strong immune pressures drive the establishment of a few escape variants, which then dominate the viral population surviving the bottleneck event. [23]	No support; suggests individual-level selection across clones. (none)
RNA: Flaviviridae	hepatitis C	Diversity increases but unknown if specific synergistic interactions leading to increased fitness gains among viruses. [24]	Increasing diversity is a precondition for social heterosis. (O2)
RNA: Flaviviridae	West Nile	Mutations occur and accumulate non-randomly across genomes. [25]	Suggestive for specific social genome evolution. (I7)
RNA: Flaviviridae	Zika	Continued passaging in cell culture leads to diverse variants with selection for mutations in NS1 and the E protein. [26]	Suggestive of selection converging on social genome. (I7)
RNA: Orthomyxoviridae	influenza	Patients with severe symptoms had a higher number of different viral haplotypes. [27]	Diverse groups have higher productivity. (I2)
RNA: Orthomyxoviridae	influenza	Intrahost diversity as a whole and on a specific set of gene segments increased with severity of outcome. [28]	Genetic diversity is important at specific loci (social genome), and such groups have higher productivity. (I2,I7)
RNA: Orthomyxoviridae	influenza	High genetic diversity during human infections. Overrepresentation of variants in reported sequences of H5N1 viruses in humans and increases in proportions of several variants over time. [29]	Increasing diversity is a precondition for social heterosis. Predictability in how diversity increases supports social genomes. (O3,I7)
RNA: Picornaviridae; Aphthovirus	foot and mouth	Rapid increase in viral diversity, multiple shifts of dominant viral haplotype during the early and transitional phases of infection, whereas few shifts occurred during persistent infection. [30]	Consistent with social heterosis predictions on genetic diversity with a stable social genome. (I4)
RNA: Picornaviridae; Aphthovirus	foot and mouth	RNA genomes evolve towards segmentation within infected cells. Strong selective pressure to favour complementary co-infection. [31]	More supportive of a rescue effect rather than social heterosis complementary interaction. (none)
RNA: Picornaviridae; Aphthovirus	foot and mouth	*In vitro* viral growth and fitness finds VP1–31G confers growth and a fitness advantage in human neuronal cells, whereas VP1–31D confers enhanced replication in colorectal cells. [32]	Different clones have different ‘tools’ for succeeding in across environments. (I2)
RNA: Picornaviridae; Aphthovirus	foot and mouth	Co-infection with multiple quasi-species (including polyomavirus, foot and mouth, and vesicular stomatitis) correlate with high viral productivity and group selection. [33]	Diverse groups have higher productivity and distribute in ways that allow group-level selection. (O3,O5)
RNA: Picornaviridae; Aphthovirus	foot and mouth	Narrow passage bottlenecks result in immediate and substantial increases in observed variant frequencies, but with no observed positive selection. Passaging large populations results in a slower, more gradual increase in observed variant frequencies. [34]	Low genetic diversity in transmission requires time for mutation to increase diversity; larger founding populations may transmit an intact social genome. (O4)
RNA: Picornaviridae; Aphthovirus	foot and mouth	Shows that extensive genetic diversity evolves quickly. [35]	Increasing diversity is a precondition for social heterosis. (O2)
RNA: Picornaviridae; Enteroviruses	polio	Co-infection common, 1/1300 viruses are a recombinant (= restores function in defective genomes). Bacteria promote co-infection (benefit to bacteria?). [36]	Genetic diversity is a precondition for social heterosis. Effects may be more rescue and mutualism than social heterosis. (O2)
RNA: Picornaviridae; Enteroviruses	polio	Viral mutation rates have evolved to be higher as a result of selection for viruses with faster replication kinetics. Suggests more rapid production of mutants, rather than genetic diversity, is the important factor. [37]	Shows the mutation rates that produce high genetic diversity may be an evolved, adaptive trait. (O1)
RNA: Picornaviridae; Enteroviruses	polio	High-fidelity mutants have reduced growth in mice and are outcompeted by low-fidelity strains. Mutations do benefit viral populations, especially in complex environments. [38]	Suggests that virus replication could have higher fidelity, but such traits are not favoured. Viruses selected to produce high diversity populations. (O1–3)
RNA: Picornaviridae; Enteroviruses	polio	A genetic bottleneck is difficult to overcome, requiring orders of magnitude increase in viral inoculum to allow representation of all or most members of the infecting pool. [39]	Host-to-host transmission of intact social genomes may be difficult. Hence, they are required to re-evolve with each transmission. (O4)
RNA: Picornaviridae; Enteroviruses	polio	Ribavirin (a mutagen) resistant viruses are less adaptable, more susceptible to antiviral drugs, and are highly attenuated *in vivo*. [40]	Viruses could easily evolve more reliable copying but it is deleterious. (O1)
RNA: Picornaviridae; Enteroviruses	polio	Poliovirus carrying a high-fidelity polymerase replicates at wild-type levels but generates less genomic diversity and is unable to adapt to adverse growth conditions. [41]	Suggests that virus replication could have higher fidelity, but such traits are not favoured. Consistent with selection occurring at group or population levels rather than on individual variants. (O1,O5)
RNA: Retroviridae	AIDS	After 10 large population passages, the viral populations showed an average increase of fitness, although with wide variations among clones. [42]	Variability and fitness relationship as expected with social heterosis. (I5)
RNA: Retroviridae	AIDS	Diversity accumulated at a 30-fold higher rate in recently infected individuals than patients with chronic infection. HIV populations are slow to change in chronic infection. [43]	Suggests that long-term infections have evolved or are converging to a stable social genome. (I6)
RNA: Retroviridae	AIDS	Genetic algorithm model reproduces multiple characteristics of HIV evolution through to immune system collapse. [6]	Social heterosis and social genomes predict multiple features of HIV infection progression. (I1–6,O1–7)
RNA: Paramyxoviridae	measles	In humans, MeV spread relies on entire block genome transmission, and that genomic diversity is instrumental for rapid MeV dissemination across hosts. [44]	Suggestive of selection converging on social genome and its genetic diversity increases productivity. (O3,I7)
RNA: Paramyxoviridae	mumps	Changes in the level of genetic heterogeneity at specific genome sites can have profound neurovirulence phenotypic consequences: either selection of one nucleotide variant at positions where the starting material exhibited nucleotide heterogeneity or the evolution of an additional nucleotide to create a heterogenic site. [45]	Genetic diversity is important at specific loci (social genome), and such groups have higher productivity. (O3,I7)
RNA: Paramyxoviridae	BRSV	Non-synonymous mutations map preferentially within the two variable antigenic regions, close to a highly conserved domain. BRSV populations may evolve as complex and dynamic mutant swarms, despite apparent genetic stability. [46]	Genetic diversity is important at specific loci (social genome), and such groups have higher productivity. (O3,I7)
RNA: Rhabdoviridae	rabies	Selection favours different virus variants in different tissues. [47]	Different clones have different ‘tools’ for succeeding in across environments (although individual-level selection may dominate at a given site). (I2,I5)
RNA: Rhabdoviridae	vesicular stomatitis	Found complementary fitness for a deleterious mutant with weak or no frequency dependence. [48]	Lack of frequency dependence a prediction of social heterosis, but effect may be more rescue than complementation. (I6)
RNA: Rhabdoviridae	vesicular stomatitis	Mutant viruses that can escape from interferon provide the same benefit to non-mutant group mates. Mutants have lower replication and are maintained by group selection. [49]	Differential group-level benefits by genotype, with group-level increase in replication. Group structure maintains diversity. (O3,I5)
RNA: Coronaviridae	MERS	Intrapatient heterogeneity was highest in a super-spreader. There was a tight correlation between the frequencies of the two variants, suggesting a combination of variants as the unit of selection. [50]	Genetic diversity correlates with productivity. Interclone dependence suggests a social genome. (O3,I2)
RNA: Hantaviridae	hanta (virion packages 3 strands)	Ribavirin's mechanism of action lies in challenging the fidelity of the hantavirus polymerase, which causes error catastrophe. [51]	Suggests that virus replication could have higher fidelity, but such traits are not favoured. (O1)
RNA: Togaviridae	chikungunya	Populations with greater genetic diversity can cause more severe disease and stimulate antibody responses with reduced neutralization of low-diversity virus populations *in vitro*. [52]	Genetic diversity correlates with productivity. Interclone fitness effects suggest a social genome. (O3,I2)
RNA: Cystoviridae	Bacteriophage	Fitness distribution of mutants differed between the two populations derived from a common ancestral phage. The more diverse population contained many more advantageous mutants. [53]	Social heterosis predicts differ trajectories in the creation of diversity across hosts. More diverse populations, more likely to show social heterosis. (O1,O4)

**Table 3.** Summary of results in cancer studies relative to evidence for social heterosis. The support is categorized relative to specific overall predictions (O) or about clonal interactions (I) from table 1.

cancer	results [study]	support for social heterosis (categories from table 1))
breast	Circulating tumour cell (CTC) clusters have 23- to 50-fold increased metastatic potential and abundance of CTC clusters denote adverse outcomes. Heterogeneity within CTC clusters is closely related to cellular-scale genetic heterogeneity within tumours. [54]	Heterogeneous groups of cancer cells produce more aggressive tumours. (O3)
breast	Heterotypic interactions between epithelial subpopulations are critical to collective invasion of breast tissue. [55]	Genetically diverse cancer populations are more invasive. (O3)
breast	Polyclonal seeding by cell clusters in a mouse model of breast cancer accounts for >90% of metastases. Tumour cell clusters induced greater than 15-fold increase in colony formation *ex vivo* and greater than 100-fold increase in metastasis formation *in vivo*. [56]	Heterogeneous groups of CTCs are more successful at metastasis. (I1)
breast	Two subclones are required for efficient tumour propagation in mice. When biclonal tumours are challenged by withdrawing a clone, they recruit heterologous cells to restore tumour growth. [57]	Genetically different clones form complementary pairings. These subclones are the most common, suggesting their presence requires group-level selection. (I2)
breast	Plakoglobin, as a key component in cell adhesion, can promote collective metastasis. Tumour emboli were associated with a high frequency of metastasis. [58]	Heterogeneous groups are more successful at metastasis. (I1)
breast	Minor subclones in breast cancer cooperate to promote metastatic spread and generate polyclonal metastases composed of driver and neutral subclones. [59]	Genetically different clones form synergistic pairings. Complementary subclones are the most common, suggesting their presence requires group-level selection. (I2,I6)
breast	Patients with a continuous presence of apoptotic or clustered CTCs after systemic therapy initiation had worse prognosis. [60]	Heterogeneous CTCs produce more aggressive tumours. Diverse tumours better defend against immune systems. (I1)
breast	Patients with CTC clusters had significantly worse survival compared with patients without clusters. [61]	Heterogeneous CTCs produce more aggressive tumours. (I1)
breast	Polyclonal CTC clusters were less frequently detected but more metastatic than single CTCs. [62]	Heterogeneous CTCs produce more aggressive tumours. (I1)
breast	The co-injection and co-growth of two different breast cancer cell line subclones resulted in increased efficiency of migration and invasion. A subclone on its own was unable to form lung metastases. [63]	Genetically different clones form and require complementary pairings to succeed. (I2)
breast	Tumour growth in a mouse xenograft can be driven by a minor cell subpopulation, which enhances the proliferation of all cells within a tumour by overcoming environmental constraints, but can be outcompeted by faster proliferating competitors, resulting in tumour collapse. [64]	Genetically different clones form complementary pairings. These subclones are the most common, suggesting their presence requires group-level selection. (I2,I6)
breast	Tumours with high heterogeneity correlated with worse survival. High diversity tumours had less activation of immune response and decreased infiltration of anti-tumour cytotoxic and helper T-cells. [65]	Heterogeneous CTCs produce more aggressive tumours. Diverse tumours better defend against immune systems. (I1)
breast	Mammary non-metastatic carcinoma cell lines metastasized to the lungs only when co-injected into mice with highly metastatic clones. [66]	Genetically different clones form and require complementary pairings to succeed. (I2)
breast	CTC clusters are more pronounced in patients with inflammatory breast cancer, the most aggressive form of breast cancer with the poorest survival. [67]	Heterogeneous CTCs produce more aggressive tumours. (I1)
breast	Found a high degree of genetic heterogeneity both within and between distinct tumour cell populations. Some tumours were markedly different between the *in situ* and invasive cell populations. [68]	The first expressions of diversity in evolving populations show stochastic differences in mutations. (O2)
breast	Patient-derived tumour xenografts can recapitulate the original intratumour genetic heterogeneity, the genomics and response to treatment, and a loss of heterogeneity in metastases compared with primary tumours. [69]	Across different assemblages in the same patient similar social genomes eventually reappear. (I7)
breast	In mice, breast tumour clones display specialization in dominating the primary tumour, contributing to metastatic populations or showing tropism for entering the lymphatic or vasculature systems. [70]	Different clones have different ‘tools’ for succeeding in their environment. (I3)
breast	Mesenchymal CTCs occur as both single cells and multicellular clusters that express a variety of characteristics. [71]	Heterogeneous CTCs vary in their characters. (O1)
colorectal	Diverse populations of cancer clones are arranged in small, intermingling areas, resulting in a variegated pattern of diversity. [72]	Local communities fit the structured deme model for group selection. (O5)
colorectal	Tumour mixed-cell doublets and mixed-cell clusters were detected in 22 of 24 colorectal patients. Chemotherapy does not destroy all of the CTCs. [73]	Group-level selection for the most effective social genome against chemotherapy. (O5)
colorectal	All samples from colorectal cancer patients found a multicellular origin of metastasis, ranging from 3 to 17 cells. This may explain dissimilarity in drug responses between tumours. [74]	Group-level selection for the most effective social genome against chemotherapy. (O5)
colorectal	The CTC clusters contain parenchymal cancer cells together with immune cells, cancer-associated fibroblasts, tumour stroma and platelets that reflect the heterogeneity of their primary tumour. [75]	Across different assemblages in the same patient similar social genomes eventually reappear. (I7)
colorectal	The presence of polyclonal CTCs positively correlates with poor prognosis. [76]	Heterogeneous CTCs produce more aggressive tumours. (I1)
oesophageal	A high degree of intratumoural heterogeneity was identified in 41 patients, and clonal populations coexisting at submillimetre distances associates with worse survival. [77]	Local communities are diverse and fit the structured deme model for group selection. (O5)
oesophageal and breast	The extent of clonal diversity predicts the probability of malignant progression in oesophageal squamous cell carcinoma and breast cancer. [78]	Heterogeneous groups of cancer cells produce more aggressive tumours. (O3)
oesophageal	Across 18 individuals, in 90% multiple subclones spread very rapidly from the primary tumour. Subclones spread to multiple organs of different types and evidenced selection *in situ*. [79]	Local communities are diverse, spread as diverse groups, and fit the structured deme model for group selection. (O5,I1)
glioblastoma	Clones depend on cell–cell contact to coordinate growth rates and protect slow-growing clones. Two drug-sensitive clones develop resistance de novo when cooperating. [80]	Genetically different clones form and require complementarity to develop drug resistance. (I2)
glioblastoma	Clonal evolution trees indicated higher heterogeneity in the relapse tumour. Aggressive chemotherapy and radiation may have applied selective pressure for tumour clonal evolution. [81]	Local communities are diverse and fit the structured deme model for group selection. (O5)
hamster cheek pouch carcinoma	Epithelial–mesenchymal transition. (EMT) and non-EMT cells cooperate in the spontaneous metastasis process. EMT cells appear responsible for degrading the surrounding matrix. Non-EMT cells then enter the bloodstream and establish in secondary sites. [82]	Genetically different clones form and require complementary pairings to succeed. (I2)
leukaemia	Murine leukaemia with four antigenically different subtypes of leukaemia cells had severe negative impacts on therapeutical effect of monoclonal antibodies. Intratumoural heterogeneity with respect to these antigens was also found in humans. [83]	Genetic diversity observed in mouse and human cancers and correlates to negative outcomes. (O3)
leukaemia	10 of 12 chronic lymphocytic leukaemia cases treated with chemotherapy (versus 1 of 6 without treatment) underwent clonal evolution, predominantly involving subclones with multiple driver mutations that expanded over time. The presence of subclones predicts adverse clinical outcomes. [84]	Group-level selection for the most effective social genome against chemotherapy. (O5,17)
leukaemia	Clonal progression is the key feature of transformation to invasiveness. The clinically predominant invasive clone first arises in the non-invasive stage. [85]	It takes some time for complementary mutations to arise to create invasiveness. (O4)
leukaemia: lymphocytic	Patients carried multiple STAT3 mutations, which were located in different lymphocyte clones. The size of the mutated clone correlated well with the degree of clonal diversity. [86]	Genetic diversity observed in cancer progress and correlated with increased cancer cell presence. (O3)
leukaemia	Negative outcomes correlate with cellular genetic diversity. Mutations affecting specific kinase signalling pathways were enriched in progressive relative to non-progressive patients. [87]	Genetic diversity correlates with negative outcomes. The same mutations and combinations observed repeatedly. (O3, I5 and 7)
lung, colon and breast	CTCs composed of multiple clones of cancer cells or composed of cancer and non-cancer cells are more likely to result in poor outcomes. [88]	Genetically diverse cancer populations are more invasive. (O3)
lung	Cellular and morphological heterogeneity correlates to prognostic failure of drugs approved for non-small cell lung cancer. [89]	Genetically diverse cancer populations are more invasive. (O3)
lung and brain	Metastatic cells can bring stromal components including activated fibroblasts from the primary site to the lungs. Brain metastases from lung and other carcinomas contain carcinoma-associated fibroblasts, in contrast with primary brain tumours. [90]	Different social genomes can arise in different tissues and successfully metastasize. (I5)
lung	Intratumour heterogeneity mediated through chromosome instability is associated with increased risks of recurrence or death. [91]	Genetically diverse cancer populations are more invasive. (O3)
lung	Polyclonal seeding of metastases occurs in a murine model of small cell lung carcinoma. [92]	Genetically diverse cancer populations are more invasive. (O3)
lung	Long-term surveillance indicated that the presence of pre-operative CTC clusters predicts poor prognosis. [93]	Heterogeneous CTCs produce more aggressive tumours. (I1)
lymphoma: cutaneous	Multiple neoplastic clones detected in the peripheral blood in all examined patients. [94]	Populations within patients are genetically diverse and variable across locations. (O5)
melanoma	In a zebrafish-melanoma xenograft model, inherently invasive cells, which possess protease activity and deposit extracellular matrix, co-invade with subpopulations of poorly invasive cells. [95]	Different clones have different ‘tools’ for complementary success in their environment. (I2)
melanoma	Multiple lesions within a patient were genetically divergent, with one or more tumours harbouring unique somatic mutations. However, certain mutations present in the first metastasis were always preserved in subsequent metastases. [96]	Diversity evolves in different pattern across genomes, where some mutations are essential for the social genome. (I5,I7)
melanoma	Solitary melanoma cells injected into target mouse liver fail to initiate growth. [97]	Clones require complementary pairings to succeed. (I2)
melanoma	Clones isolated from mouse melanoma cell lines show extensive cellular heterogeneity and the presence of subpopulations that have widely differing metastatic abilities. Members of polyclonal subpopulations somehow interact with one another to ‘stabilize’ their relative proportions. [98]	Evidence for a stable social genome. (I3)
myeloma	Throughout progression novel mutations correlate with subclonal evolution from clone reservoirs. Subclones can have novel mutations for drug sensitivity. [99]	Diversity increases with cancer progression. Clones vary in treatment resistance. (O2,I5)
myeloma	Different phenotypic myeloma subclones may frequently show unique cytogenetic and clonogenic features. [100]	Populations are genetically diverse and clones vary in their growth potential. (O1,I5)
pancreatic	The level of circulating tumour microemboli is an independent negative predictor of poorer overall survival and progression-free survival. [101]	Heterogeneous CTCs produce more aggressive tumours. (I1)
pancreatic	In a mouse model, precursor lesions exhibit high heterogeneity but diversity decreases during premalignant progression. A significant fraction of metastases, however, are polyclonally seeded by distinct subclones. This suggests heterotypic interactions between tumour subpopulations contribute to metastatic progression. [102]	Genetic diversity is important but in certain combinations. Diversity increase selected against in the presence of a social genome. (O6,I2)
pancreatic	Clonal populations in metastases are represented within the primary carcinoma, but are genetically evolved from the original parental, non-metastatic clone. It may take a decade between the initiating mutation and appearance of the parental, non-metastatic founder cell, 5 more years for acquisition of metastatic ability, and an average of 2 years to death. [103]	Social heterosis may take a long and variable time because complementary mutations must both arise and be spatially close. (I4)
prostate	Metastases can spread via polyclonal seeding, as observed in patients with prostate tumours. [104]	Genetically different clones form complementary pairings. (I2)
prostate	The invasiveness of the cancer stem cell-enriched subpopulation is enhanced by the non-cancer stem cell population, accelerating metastatic spread. [105]	Heterogeneous groups of cancer and non-cancer cells are more successful at metastasis. (O3)
multiple cancer types	Early oncogenesis is characterized by mutations in a constrained set of driver genes, and specific copy number gains. Timing analyses suggest driver mutations often precede diagnosis by many years, if not decades. [106]	Some mutations are essential. Social heterosis may take a long and variable time because complementary mutations must both arise and be spatially close. (O4,I7)
multiple cancer types	CTCs can migrate from secondary metastatic tumours back to the primary tumour, increasing its diversity and growth rate (self-seeding). [107]	Heterogeneous groups are more successful at metastasis. (I1)
multiple cancer types	Oncogenic mutations frequently are present as subpopulations within tumours, rather than as the pure mutant populations expected to develop from a single initiated cell. [108]	It takes some time for complementary mutations to arise to create invasiveness. (O4)
multiple cancer types	Within seven types of untreated epithelial cancers, primary tumours are genetically homogeneous with respect to functional driver-gene mutations in metastases. [109]	Diversity evolves in different pattern across genomes, where some mutations are essential for the social genome. (I7)

### 3.1. Viral literature

Across virus studies, RNA viruses provide considerably more evidence for social heterosis than do DNA viruses (table 2). RNA viruses produce a low-fidelity polymerase, resulting in high mutation rates [110,111]. Over the course of an infection in a host, a single infecting clone can rise to large number of new clones that vary in one or more mutations from the original genotype (i.e. forming a quasi-species population). The genetically related variants often centre around a consensus sequence with their relative frequencies fluctuating over time [25,110]. Diversity may be inherited between hosts via co-packaging of genomes into single viral units or by a single infecting strain that rapidly re-establishes diverse populations after a transmission bottleneck [110,112].

For many RNA viral infections, higher diversity is correlated with increased virulence and larger viral load [111]. Variants may differ in biological characteristics which affect adaptive potential, such as capacity to escape the host immune system and resist antiviral or antibody treatments [24,111]. Interactions among genetically diverse variants can potentially contribute to replication rate, thermal stability, interferon release, entry and exit in cells, fighting neutralizing antibodies, antiviral resistance or other aspects of viral fitness [110]. Synergistic interactions at the population level can also influence burst size, viral load and chronicity of infection [24]. The presence of certain variants in a population, even low-frequency ones, can play a large role in infection severity [27].

Certain RNA viruses provide stronger evidence for social heterosis compared with others (table 2). For example, hepatitis B, influenza, measles, MERS and chikungunya viruses present strong evidence in support of social heterosis. Hepatitis B exhibits rapid diversification at specific loci during early infection, which slows down as the disease progresses potentially due to the elimination of non-complementary genomes [19]. In Influenza infections, groups with greater genetic diversity at particular loci were more productive compared with homogeneous groups, suggesting the presence of synergistic interactions between such loci [28]. Measles viruses carry multiple genomes which code for different functional proteins that synergistically interact to increase viral spread in culture [44]. Successful measles infections rely on *en bloc* transmission to a new host, where multiple genomes were transmitted at once due to diversity being a necessity for rapid dispersal [44]. Individuals infected with the highest level of diversity of MERS viruses became ‘super spreaders’. There is a strong correlation between the co-occurrence of certain variants, supporting selection beyond the individual level [50]. Aggregates also formed to co-infect cells and create chimeric plaques [36]. In Chikungunya infections, genomic diversity correlated with higher viral productivity and disease severity in line with social genome theory [52].

### 3.2. Cancer literature

In cancer, the evidence for social heterosis is strongly suggestive (table 3). Most tumours are heterogeneous ecosystems of interactive cell populations [74,113]. In many cancers, increased tumour diversity correlates with faster growth rate, higher malignancy and higher resistance to therapy (table 3). Polyclonal seeding occurs when CTC clusters collectively invade instead of single cells and are associated with improved survival in the bloodstream, higher colonization success in distant tissues and promotion of stem-like behaviour [75,88,114,115]. Clusters may have up to a 50-fold higher rate of metastasis compared with single cells [63,116]. While cell-intrinsic properties and specific driver mutations lead to the epithelial to mesenchymal transitions that precede metastasis, cells with these mutations alone may not survive migrating in the circulatory system to establish new lesions at distant organs [106,115,117]. Thus, even with strong selective pressures for driver mutations, tumour diversity is maintained throughout cancer progression [64,115]. Indeed, interactions between tumour subclones contribute to faster and more efficient metastases in many cancers [74,88,114,118].

Several cancers show strong evidence for social genomes and patterns of diversity which correlate with increased aggression of tumours (table 3). Breast cancers exhibit CTC clusters composed of genetically distinct subclones which are associated with worse survival for patients compared with those who lack CTC clusters [54,56,59,61]. Similarly, colorectal, pancreatic and lung cancers also exhibit the presence of diverse CTC clusters which are correlated with poorer prognosis [76,93,101,102]. The association between increased diversity and tumour aggression is also seen in oesophageal cancers [77,78].

## 4. Discussion

A pattern from ecological theory commonly found in natural communities is the competitive exclusion principle [119–126]. Briefly stated, two species cannot coexist over time in the same ecological niche, when they are competing for the same set of essential resources. The better competitor in the majority of cases will drive the poorer competitor to extinction. Thus, within rapidly mutating quasi-species swarms of RNA viruses or cancer cell clone lineages, some genotypes should have an advantage at exploiting their surroundings and replicating. Over time, natural selection would be expected to favour the better adapted clones, and for such ‘superclones’ to proportionally dominate their respective populations and reduce the overall genetic diversity of the population.

Inconsistent with the rise of one or a few dominant clones, however, the growth and metastasis of tumours and the virulence of viral infections often correlates with increasing genetic diversity, *per se*. The initial evolution and maintenance of clonal diversity could result from rescue effects. Genetically defective clones, unable to synthesize a vital product, can parasitize the output of viable ones in their vicinity (e.g. [31]). In turn, such defective clones may provide a reciprocal benefit to functional or wild-type clones by indirectly helping in escaping attack by immune systems. Overall burgeoning diversity of both functional and defective creates ‘too-many-targets’ for the host's immune system, and the defences become overwhelmed. Should this happen, rescue effects and ineffective immune system control might weaken selection in favour of any particular genotype of pathogen or cancer cell line. This would create correlations between increasing genetic diversity and negative health outcomes.

Our goal is not to evaluate how often superclone or too-many-targets models explain disease progression. Instead, we propose a third model that incorporates the genetically diversifying mechanisms evident in RNA virus and cancer cell replication, with the possibility that certain genetic combinations interact synergistically in complementary ways with each other. This requires social heterosis—the process in which groups of diverse and complementary genotypes can be more productive than groups composed of any single genotype [4–6]. Such fortuitous combinations will create a more complex genetic entity: the social genome. It is the within-host appearance and evolution of social genomes that can increase virulence in an infection, transition an acute condition to a chronic one and contribute to the metastatic tendencies of cancers.

The social heterosis model makes a number of predictions about overall patterns of genetic diversity and genotypic or phenotypic interactions within a host (table 1). A review of the literature finds multiple studies with observations that match specific predictions across a variety of virus families (table 2) and multiple types of cancers (table 3). Some of the supporting observations are general and could occur in the absence of social heterosis, such as pathogen populations being composed of multiple genotypes, quasi-species swarms of RNA viruses appearing because of low fidelity in replication, or cancer tumours becoming genetically heterogeneous because of genomic instability. The social heterosis model, however, also makes more specific predictions that would differ from patterns generated by either superclone or too-many-targets models. Thus, in a number of studies, only the social heterosis model is consistent with observations of genetic diversity initially increasing within a host, but then plateauing or declining with chronic or severely negative health outcomes; genetically diverse CTCs driving cancer metastasis; clear evidence of complementary interaction between genetically distinct clones (particularly for genetic variants at the same loci); weak or absent frequency-dependent selection as genetic diversity increases in populations; and convergent evolutionary outcomes across hosts for particular mutations or allelic combinations.

If social heterosis occurs and is an important component for viral diseases and cancers, it fundamentally alters our perceptions of the conflict between disease and host. For example, the HIV genome is composed of only nine genes, yet it effectively manipulates hundreds of different human proteins [127]. Consider, however, that during the asymptomatic phase of an HIV infection, the virus is creating a polyploid entity that will compose the complementary social genome. The immune system being thereafter overwhelmed and collapsing when facing a cloud of synergistic, interactive genomes becomes less puzzling. Similarly, a typical cancer cell with a degraded or damaged genome would seem to stand little chance of survival by itself against a vigilant immune system [128]. However, groups of cancer cells, each with a different ‘tool’ to fend off immune responses and to manipulate non-cancerous cells in the immediate vicinity, become a formidable foe capable of growing and propagating throughout a body. Furthermore, to the degree that social heterosis is involved, then negative health outcomes are being evolutionarily driven by group-level selection processes. This may require a different way of thinking in terms of therapeutic approaches [4,41,46,116,129–131].

The fact that social heterosis can occur and social genomes may arise analogously in both quasi-species swarms of RNA viruses and cancer tumours leads to interesting evolutionary comparisons. Viruses and their hosts have been in a several billion-year coevolutionary arms race from perhaps the origin of life on Earth [132]. This arms race, however, is evolutionarily balanced. Mutations in a viral population that increase infectivity and the ability to use host resources are under positive selection. At the same time, any host mutation that helps stave off viral infection or ameliorates one should also be selectively favoured. By contrast, however, every new cancer literally replicates the origin of the first virus. There is no billion-year continuous history of interaction. Therefore, all evolutionary arms races begin in an unbalanced state. The host has billions of years of ancestors that either never got cancer or at least survived theirs long enough to reproduce. Whatever genetic constitution allowed the ancestor to win was passed to its descendants. Any favourable mutations that produce genetically successful cancers, however, die with the host. Neither winning nor losing cancers leave any offspring. With such a long evolutionary asymmetry in favour of beating cancer, it is somewhat paradoxical that cancer both exists and often wins the competition against their hosts' non-cancerous cells.

The evolutionary advantages for social heterosis may also help explain a dichotomous distribution in viral genomics. For example, viruses that attack single-celled organisms have little opportunity to develop and evolve quasi-species swarms within the attacked cell. Therefore, suggestive of the importance of being able to make genetically diverse populations, across 47 recognized families of RNA viruses, only a few species in the families Leviviridae and Cystoviridae are known to be bacteriophages [133]. Instead, most RNA viruses attack multicellular eukaryotic species. Within eukaryotes, RNA virus reproduction is predominantly lytic (replicating rapidly within cells and killing them) rather than lysogenic (incorporating into a host's DNA and replicating with the host). Only retroviruses (four families in the order Ortervirales) make DNA copies of their RNA that can be inserted in host genomes. HIV is the best studied retrovirus and although it may have lysogenic capabilities, its lytic life history produces quasi-species swarms that correlate to immune system collapse [6]. Increasing the number of reproductive generations along with a high mutation rate allows quasi-species swarms to appear in the longer-lived multicellular hosts [41,134]. The fact that RNA replication is highly error prone and rapidly produces genetic diversity may be actually a vital adaptation whose rate is optimally tuned. It is possible to manipulate viruses into replicating with less error, but such manipulations make the viruses less successful rather than more so [37,38,40,135].

In terms of treatment regimes for specific viral diseases, it may, therefore, be an important consideration as to whether the virus has an RNA or DNA genome. For example, if replication error rates are too high, populations of viruses could produce so many defective genotypes that the population no longer has enough viable individuals to sustain itself. The population crosses a threshold into a state of ‘error catastrophe’ [136]. The very high mutation rates of RNA viruses suggest they are pushing close to where error catastrophe results in population extinction. Although this suggests that further increasing the mutation rate of a targeted RNA viral disease might have therapeutic value, such a strategy could also be counter-productive [137,138]. For example, when RNA viruses are rapidly serially transmitted *in vitro* in the presence of mutagens, error catastrophe can occur and result in population collapse [139,140]. Unfortunately, these methods probably fail to transmit any existing social genome through genetic bottlenecks and force a reset of social heterosis to its initial stage. In comparison, the results of increasing the mutation rate of a chronic, existing infection have little positive effect and even can potentially increase negative outcomes [141,142].

Given that viruses have had a long evolution history towards solving the error catastrophe problem, it may be instead more effective to push their populations in the opposite direction. Hence, increasing replicative fidelity and decreasing recombination rates might be an effective strategy against RNA diseases. The reduced error rates and genetic exchange could increase the time, or prevent entirely, the evolution of a stable social genome. Indeed, an RNA virus that is unable to mutate or recombine at the onset of an infection might be quickly eliminated by host immune responses. In a simulated HIV infection, Nonacs & Kapheim [6] found that reintroducing a wild-type genotype that was competitively superior at the individual level of selection, disrupted any social genome that had formed and reduced viral loads. This suggests that a quasi-species swarm could be susceptible to being competitively constrained or eliminated by introducing a competing clone with high fidelity in replicating its genome, but without across-clone complementation.

The fact that many cancers increase in frequency with organism age and across species strongly suggests that organism-level susceptibility to cancer is also an evolvable entity [143]. From the point of view of a tumour, however, it appears as a de novo organism in every patient, with the cancer's success or failure being entirely specific to only that patient. Thus, studying how the overall human immune system changes over time and compares with other more cancer-free species might be useful and informative in an evolutionary medicine context. For example, the taxonomic distribution of cancer across species strongly suggests it is far more prevalent in groups that strictly separate cell lineages into either the germ line (the gonads) or non-gamete-producing somatic tissue and organs [143]. Taxonomic groups (e.g. cnidarians) in which all or most cells retain their totipotency to switch to gamete production are far less likely to develop cancer. This cancer deficit occurs even though oncogenes (or at least their evolutionary precursors) are present in these groups [144].

Cancer cells themselves are proposed to be the equivalent of evolutionarily non-cooperative cheaters—as cells that in a sense rebel against imposed sterility and low growth [145,146]. Cheaters, therefore, are paradoxically more common in taxa that in the course of normal ontogeny more strongly restrict and limit the range of cell lineage developmental trajectories. Furthermore, within individuals, more aggressive tumours with higher variability tend to arise in organs with apparently stronger anti-cancer defences in comparison with other tissues [147]. Such patterns are counterintuitive. Stronger controls that limit what function a cell can express ought to make cancers less likely rather than more so. Resolving why this apparent evolutionary paradox exists could lead to future therapeutic options.

Complementation promoting tumour growth and creation of the tumour microenvironment can arise from cell–cell signalling, i.e. with the secretion of growth factors, hormones, cytokines, extracellular matrix regulators, tumourigenic fibroblasts and other factors affecting angiogenesis, oxygen metabolism and defence against the immune system [115,148–150]. Due to the complexity of the tumour microenvironment, it is unlikely for single genotypes to be able to create the plasticity required to create and maintain a completely hospitable environment [59,115,130,151]. Via complementation among heterogenous genotypes, however, it is possible to create a system of labour division where all group members can benefit in accordance with functional social genomes.

The lack of any coevolutionary history between cancerous and non-cancerous cells, however, forces the conclusion that complementation and social genomes in cancer tumours and metastases are serendipitous by-products of unstable and mutable genomes. Targeting single clones for therapy often fails to predict the properties of a mixed population [152]. Therefore, incorporating the ideas of social heterosis could be valuable in improving the treatment of cancer, but this requires thinking in general terms about the process. One such aspect is that a social genome can be a network of interdependent genes. Some mutations or clonal variants may perform redundant functions. Thus, attacking or eliminating a particular clone, even if the mutation it carries is associated with increased malignancy, may have no effect on disease progression. Instead, there may be a ‘keystone’ genetic change within a social heterosis network—which if eliminated—severely impacts a tumour's ability to grow and metastasize. Social heterosis predicts that a clonal line carrying such a keystone needs to be common enough to be a regular member of a CTC or tumour, but it need not be the most common cell type or exhibit any significantly measurable frequency-dependent effects. Unfortunately, such keystone changes may very well differ across patients and, therefore, not lead to a general treatment. However, some types of cancers may indeed have a particular pathway or keystone change that needs to convergently evolve across patients that otherwise produce genetically unique tumours at a population level of cancer cells. To the degree that any such changes are seen to be shared across patients, the clonal lineages with those changes might be particularly useful for therapies to directly target.

Genetic diversity with social heterosis means targeting population heterogeneity could disrupt synergistic interactions and lessen disease progress. Quasi-species expansion in RNA viral infections could be prevented by increasing RNA polymerase fidelity, or conversely, at the earliest stages of infection pushed past their error threshold by decreasing fidelity [153–155]. Similarly, cancer drugs that induce mitotic catastrophe, disruptively intercalate DNA or affect genes such as TP53 that help regulate chromosomal stability could directly target tumour heterogeneity and hinder the synergistic interactions of social heterosis [156].

## Data accessibility

This article has no additional data.
