# Supplementary material for: Genetic diversity through social heterosis can increase virulence in RNA viral infections and cancer progression
Source: R Soc Open Sci. 2021 May 5;8(5):202219. doi: 10.1098/rsos.202219 (PMC8097216; doi:10.1098/rsos.202219)
Supplement: Supplementary file 1 [file rsos202219_review_history.pdf]

THE ROYAL SOCIETY
PUBLISHING

ROYAL SOCIETY
OPEN SCIENCE

Genetic diversity through social heterosis can increase virulence in RNA viral infections and cancer progression

Saba Ebrahimi and Peter Nonacs

Article citation details
R. Soc. open sci. **8**: 202219.
http://dx.doi.org/10.1098/rsos.202219

Review timeline
Original submission: 7 December 2020
Revised submission: 7 April 2021
Final acceptance: 12 April 2021

Note: Reports are unedited and appear as submitted by the referee. The review history appears in chronological order.

Review History

RSOS-202219.R0 (Original submission)

Review form: Reviewer 1

Is the manuscript scientifically sound in its present form?
Yes

Are the interpretations and conclusions justified by the results?
Yes

Is the language acceptable?
Yes

Do you have any ethical concerns with this paper?
No

Have you any concerns about statistical analyses in this paper?
No

Recommendation?
Accept with minor revision (please list in comments)

Reports © 2021 The Reviewers; Decision Letters © 2021 The Reviewers and Editors; Responses © 2021 The Reviewers, Editors and Authors. Published by the Royal Society under the terms of the Creative Commons Attribution License http://creativecommons.org/licenses/by/4.0/, which permits unrestricted use, provided the original author and source are credited

Comments to the Author(s)
Absolutely great paper overall. Novel, fascinating, well researched and well written.
I offer some suggestions for improvement.

-listing predictions of the model more explicitly would be useful

-listing applied implications more explicitly would be useful

-the model should apply to some forms of cancer (solid) but not so much to others (blood); please specify

-why is the model not discussed in the context of pathogenic bacteria (eg with cross-feeding)

-explain quasispecies briefly; most readers will not know about them

-what sort of ENVIRONMENTS (ecological conditions) select for social heterosis most, least, under the model you describe. After all, in social insects, environments are of central importance

-how is genetic relatedness involved in social heterosis in viruses and cancers, exactly?

-Axelrod's work on cooperation between cancer cells (PNAS) ought to be cited; plus be sure to see papers that cited it. Axelrod. DE. ,. Pienta. KJ. et al. Evolution of cooperation among tumour cells ... http://dx.doi.org/10.1073/pnas.0606053103.

-viruses may provide good models for group selection effects because the infected cells impose a spatial structure that facilitates group selection. do you agree? if so add to ms.

-the immune system is expected, as in HIV, to differentially attack the most common variants of a virus, leading to increased and maintained diversity. do you agree? if so add to ms.

The authors stated:
'Furthermore, within individuals, more aggressive tumors with higher variability tend to arise in organs with apparently stronger anti-cancer defenses in comparison to other tissues [138]. Such patterns are counter intuitive. Stronger controls that limit what function a cell can express ought to make cancers less likely rather than more so. Resolving why this apparent evolutionary paradox exists could lead to future therapeutic options'

-I do not see why this is counter-intuitive. Only strong tumors could arise and survive in well-defended organs.

- see also

Nat Microbiol
• 2019 Jun;4(6):1006-1013.
doi: 10.1038/s41564-019-0379-8. Epub 2019 Mar 4.
Social evolution of innate immunity evasion in a virus

and

Volume 22, Issue 4, 11 October 2017, Pages 437-441
Commentary
Sociovirology: Conflict, Cooperation, and Communication among Viruses

Decision letter (RSOS-202219.R0)

We hope you are keeping well at this difficult and unusual time. We continue to value your support of the journal in these challenging circumstances. If Royal Society Open Science can assist you at all, please don't hesitate to let us know at the email address below.

Dear Dr Nonacs

On behalf of the Editors, we are pleased to inform you that your Manuscript RSOS-202219 "Genetic diversity through social heterosis can increase virulence in RNA viral infections and cancer progression" has been accepted for publication in Royal Society Open Science subject to minor revision in accordance with the referees' reports. Please find the referees' comments along with any feedback from the Editors below my signature.

I apologise for the time this has been in review, which has been down to the difficulty of finding reviewers. However, we have one very positive review who raises a number of minor points.We invite you to respond to the comments and revise your manuscript. Below the referees' and Editors' comments (where applicable) we provide additional requirements. Final acceptance of your manuscript is dependent on these requirements being met. We provide guidance below to help you prepare your revision.

Please submit your revised manuscript and required files (see below) no later than 7 days from today's (ie 31-Mar-2021) date. Note: the ScholarOne system will 'lock' if submission of the revision is attempted 7 or more days after the deadline. If you do not think you will be able to meet this deadline please contact the editorial office immediately.

Please note article processing charges apply to papers accepted for publication in Royal Society Open Science (https://royalsocietypublishing.org/rsos/charges). Charges will also apply to papers transferred to the journal from other Royal Society Publishing journals, as well as papers submitted as part of our collaboration with the Royal Society of Chemistry (https://royalsocietypublishing.org/rsos/chemistry). Fee waivers are available but must be requested when you submit your revision (https://royalsocietypublishing.org/rsos/waivers).

Thank you for submitting your manuscript to Royal Society Open Science and we look forward to receiving your revision. If you have any questions at all, please do not hesitate to get in touch.

Best regards,
Lianne Parkhouse
Editorial Coordinator
Royal Society Open Science
openscience@royalsociety.org

on behalf of Professor Steve Brown (Associate Editor) and Steve Brown (Subject Editor)
openscience@royalsociety.org

Reviewer comments to Author:
Reviewer: 1
Comments to the Author(s)

Absolutely great paper overall. Novel, fascinating, well researched and well written.
I offer some suggestions for improvement.

-listing predictions of the model more explicitly would be useful

-listing applied implications more explicitly would be useful

-the model should apply to some forms of cancer (solid) but not so much to others (blood); please specify

-why is the model not discussed in the context of pathogenic bacteria (eg with cross-feeding)

-explain quasispecies briefly; most readers will not know about them

-what sort of ENVIRONMENTS (ecological conditions) select for social heterosis most, least, under the model you describe. After all, in social insects, environments are of central importance

-how is genetic relatedness involved in social heterosis in viruses and cancers, exactly?

-Axelrod's work on cooperation between cancer cells (PNAS) ought to be cited; plus be sure to see papers that cited it. Axelrod. DE. ,. Pienta. KJ. et al. Evolution of cooperation among tumour cells ... http://dx.doi.org/10.1073/pnas.0606053103.

-viruses may provide good models for group selection effects because the infected cells impose a spatial structure that facilitates group selection. do you agree? if so add to ms.

-the immune system is expected, as in HIV, to differentially attack the most common variants of a virus, leading to increased and maintained diversity. do you agree? if so add to ms.

The authors stated:
'Furthermore, within individuals, more aggressive tumors with higher variability tend to arise in organs with apparently stronger anti-cancer defenses in comparison to other tissues [138]. Such patterns are counter intuitive. Stronger controls that limit what function a cell can express ought to make cancers less likely rather than more so. Resolving why this apparent evolutionary paradox exists could lead to future therapeutic options'

-I do not see why this is counter-intuitive. Only strong tumors could arise and survive in well-defended organs.

- see also

Nat Microbiol
• 2019 Jun;4(6):1006-1013.
doi: 10.1038/s41564-019-0379-8. Epub 2019 Mar 4.
Social evolution of innate immunity evasion in a virus

and

Volume 22, Issue 4, 11 October 2017, Pages 437-441
Commentary
Sociovirology: Conflict, Cooperation, and Communication among Viruses

===PREPARING YOUR MANUSCRIPT===

Your revised paper should include the changes requested by the referees and Editors of your manuscript. You should provide two versions of this manuscript and both versions must be provided in an editable format:
one version identifying all the changes that have been made (for instance, in coloured highlight, in bold text, or tracked changes);
a 'clean' version of the new manuscript that incorporates the changes made, but does not highlight them. This version will be used for typesetting.
Please ensure that any equations included in the paper are editable text and not embedded images.

Please ensure that you include an acknowledgements' section before your reference list/bibliography. This should acknowledge anyone who assisted with your work, but does not qualify as an author per the guidelines at https://royalsociety.org/journals/ethics-policies/openness/.

While not essential, it will speed up the preparation of your manuscript proof if you format your references/bibliography in Vancouver style (please see https://royalsociety.org/journals/authors/author-guidelines/#formatting). You should include DOIs for as many of the references as possible.

If you have been asked to revise the written English in your submission as a condition of publication, you must do so, and you are expected to provide evidence that you have received language editing support. The journal would prefer that you use a professional language editing service and provide a certificate of editing, but a signed letter from a colleague who is a native speaker of English is acceptable. Note the journal has arranged a number of discounts for authors using professional language editing services (https://royalsociety.org/journals/authors/benefits/language-editing/).

===PREPARING YOUR REVISION IN SCHOLARONE===

To revise your manuscript, log into https://mc.manuscriptcentral.com/rsos and enter your Author Centre - this may be accessed by clicking on "Author" in the dark toolbar at the top of the page (just below the journal name). You will find your manuscript listed under "Manuscripts with Decisions". Under "Actions", click on "Create a Revision".

Attach your point-by-point response to referees and Editors at Step 1 'View and respond to decision letter'. This document should be uploaded in an editable file type (.doc or .docx are preferred). This is essential.

Please ensure that you include a summary of your paper at Step 2 'Type, Title, & Abstract'. This should be no more than 100 words to explain to a non-scientific audience the key findings of your research. This will be included in a weekly highlights email circulated by the Royal Society press office to national UK, international, and scientific news outlets to promote your work.

At Step 3 'File upload' you should include the following files:
-- Your revised manuscript in editable file format (.doc, .docx, or .tex preferred). You should upload two versions:
1) One version identifying all the changes that have been made (for instance, in coloured highlight, in bold text, or tracked changes);
2) A 'clean' version of the new manuscript that incorporates the changes made, but does not highlight them.
-- An individual file of each figure (EPS or print-quality PDF preferred [either format should be produced directly from original creation package], or original software format).

-- An editable file of each table (.doc, .docx, .xls, .xlsx, or .csv).
-- An editable file of all figure and table captions.
Note: you may upload the figure, table, and caption files in a single Zip folder.
-- Any electronic supplementary material (ESM).
-- If you are requesting a discretionary waiver for the article processing charge, the waiver form must be included at this step.
-- If you are providing image files for potential cover images, please upload these at this step, and inform the editorial office you have done so. You must hold the copyright to any image provided.
-- A copy of your point-by-point response to referees and Editors. This will expedite the preparation of your proof.

At Step 6 'Details & comments', you should review and respond to the queries on the electronic submission form. In particular, we would ask that you do the following:
-- Ensure that your data access statement meets the requirements at https://royalsociety.org/journals/authors/author-guidelines/#data. You should ensure that you cite the dataset in your reference list. If you have deposited data etc in the Dryad repository, please only include the 'For publication' link at this stage. You should remove the 'For review' link.
-- If you are requesting an article processing charge waiver, you must select the relevant waiver option (if requesting a discretionary waiver, the form should have been uploaded at Step 3 'File upload' above).
-- If you have uploaded ESM files, please ensure you follow the guidance at https://royalsociety.org/journals/authors/author-guidelines/#supplementary-material to include a suitable title and informative caption. An example of appropriate titling and captioning may be found at https://figshare.com/articles/Table_S2_from_Is_there_a_trade-off_between_peak_performance_and_performance_breadth_across_temperatures_for_aerobic_scope_in_teleost_fishes_/3843624.

At Step 7 'Review & submit', you must view the PDF proof of the manuscript before you will be able to submit the revision. Note: if any parts of the electronic submission form have not been completed, these will be noted by red message boxes.

Author's Response to Decision Letter for (RSOS-202219.R0)

See Appendix A.

Decision letter (RSOS-202219.R1)

We hope you are keeping well at this difficult and unusual time. We continue to value your support of the journal in these challenging circumstances. If Royal Society Open Science can assist you at all, please don't hesitate to let us know at the email address below.

Dear Dr Nonacs,

I am pleased to inform you that your manuscript entitled "Genetic diversity through social heterosis can increase virulence in RNA viral infections and cancer progression" is now accepted for publication in Royal Society Open Science.

If you have not already done so, please remember to make any data sets or code libraries 'live' prior to publication, and update any links as needed when you receive a proof to check - for instance, from a private 'for review' URL to a publicly accessible 'for publication' URL. It is good practice to also add data sets, code and other digital materials to your reference list.

You can expect to receive a proof of your article in the near future. Please contact the editorial office (openscience@royalsociety.org) and the production office (openscience_proofs@royalsociety.org) to let us know if you are likely to be away from e-mail contact -- if you are going to be away, please nominate a co-author (if available) to manage the proofing process, and ensure they are copied into your email to the journal. Due to rapid publication and an extremely tight schedule, if comments are not received, your paper may experience a delay in publication.

Please see the Royal Society Publishing guidance on how you may share your accepted author manuscript at https://royalsociety.org/journals/ethics-policies/media-embargo/. After publication, some additional ways to effectively promote your article can also be found here https://royalsociety.org/blog/2020/07/promoting-your-latest-paper-and-tracking-your-results/.

On behalf of the Editors of Royal Society Open Science, thank you for your support of the journal and we look forward to your continued contributions to Royal Society Open Science.

Best regards,
Lianne Parkhouse
Editorial Coordinator
Royal Society Open Science
openscience@royalsociety.org

on behalf of Professor Steve Brown (Subject Editor)
openscience@royalsociety.org

Follow Royal Society Publishing on Twitter: @RSocPublishing
Follow Royal Society Publishing on Facebook:
https://www.facebook.com/RoyalSocietyPublishing.FanPage/
Read Royal Society Publishing's blog:
https://royalsociety.org/blog/blogsearchpage/?category=Publishing

Appendix A

Dear Editor,

The authors would like to thank you and the reviewers for your considered and constructive comments. They have brought about a marked improvement in the manuscript. Our point-by-point response is below (with our responses in **blue bold**):

Associate Editor Comments to Author (Professor Matthew Collins):
Comments to the Author:
Thank your for your MS. All three reviewers agree that this is a fascinating paper which should be published and all suggesting only minor changes.

I how that you would agree that the full data associated with the 87Sr/86Sr analyses should be included (e.g. in the supplementary section. These data should have 88Sr (V), 85Rb (V), 87Sr/86Sr, 87Sr/86Sr (1 standard error). Given the methods described in the manuscript for the determination of the 87Sr/86Sr data. It is best practice to include all the QC data generated with these analyses.

Apologies that this was omitted from our original submission. We have added the extended $^{87}Sr/^{86}Sr$ data to the supplementary material (as well the extended $\delta^{18}O$ data).

Reviewer #1
Overall this is an excellent manuscript based on a very sound study. The authors applied multiple isotope analysis (strontium and oxygen of dental enamel; sulfur, carbon and nitrogen of dentin collagen/+1 rib sample) to construct fairly detailed, yet admittedly incomplete, osteobiographies of eight individuals from the famous shipwreck of the Mary Rose. In its current form the manuscript fulfills the main criteria for publication in this journal (Articles should report work that is scientifically sound, in which the methodology is rigorous and the conclusions are fully supported by the data.) and as such is certainly suitable for publication. I was also very impressed by the clear structure and writing, and particularly how the authors were able to carefully yet successfully incorporate the ancestry estimation results into the broader study. On this point, the way that this potentially problematic data type is used, and the further clarification provided in the supplementary document, provides a useful template for future studies attempting to integrate isotopic data and osteological data sets with ancestry estimations. The authors generally struck a good balance between over- and under-interpretation. The study is also especially important for highlighting the presence of presence of non-Europeans in the British/European archaeological record of the late mediaeval/early modern period.

There are two main critiques of the ms, in its current, that if properly addressed would greatly improve this manuscript.
1) While the authors briefly mention the previous study of Bell et al. (2009) and the subsequent archaeological debate that this initiated (Millard & Schroeder 2010; Bell et al. 2010) there is no explicit effort made to engage in this debate nor even to compare the data in this study with that originally produced by Bell and colleagues. It is unclear why this is the case, and understandable that the authors clearly wish to emphasize their own new data.

Nonetheless, since the original study by Bell et al. included some of the same isotope systems (namely, oxygen, carbon and nitrogen) applied to the same exact skeleton collection, the complete lack of engagement with, or direct comparison to this earlier study is problematic. If the authors have reason to doubt the reliability of the data reported by Bell and colleagues, then they are obligated to be explicit about this. If not, then it really is necessary to make a clear comparison between the isotope data from Bell et al. (2009) and the isotope data reported herein (e.g. how do they compare? do the ranges overlap? are the results consistent with the previous study or differ significantly?, etc...). The data from both studies should also be plotted together in one or more figures to allow the readers to directly compare and interpret the data themselves.

We are grateful for these constructive comments and we agree that we should provide a clearer comparison with Bell et al.'s (2009) data. There are reasons for our not having done this in the original submission. We have some concerns about the comparability of the oxygen isotope data and therefore did not undertake detailed comparison initially. We appreciate that this is an odd omission so have added a caveated comparison and explained our concerns. We have added the following sentences to our discussion section to address this:

'Comparison with existing data from the Mary Rose must also be made with caution. Bell et al.'s [9] data are not directly comparable due to a different sampling methodology and the use of a NaClO pre-treatment, which has been demonstrated to make $\delta^{18}O$ values lower [114]. Once converted to $\delta^{18}O_p$ [83], the mean for the previous dataset (17.6 ±0.87‰, 1σ) is markedly lower than in this study (19.2 ±0.95‰, 1σ), although there is considerable overlap in the datasets. This difference may derive from chance sampling, as both studies relate to only a small number of individuals, or may relate to varied sampling and pre-treatment methods.'

For the carbon and nitrogen data, comparisons have been made via the biplot (Figure 4).

Also, a more explicit comment on earlier examples of African individuals recovered from (Roman) British archaeological contexts is perhaps merited (Leach et al. 2010).

We appreciate the reviewer's suggestion but do not feel that we need to refer to examples of individuals of African ancestry found in Roman contexts as this is a post-medieval study period; we believe that the reference to historical evidence for people of African ancestry in the Tudor period is sufficient.

2) As is the case with most isotopic provenance studies, the approach used is (by necessity) exclusionary. However, the manner of presenting these types of results (rather long descriptions listing all of the places where each individual could NOT have originated from for each isotope proxy) is less than ideal. In most geographic contexts there is no other option than to present the results this way. However, Britain is the most intensively and extensively mapped areas of the world for most isotope systems, and one of the few regions where high quality isoscapes exist for both strontium and oxygen isotopes (e.g. Evans et al .2012; Pelligrini et a. 2016; see also the British isotope domains dataset and online tool at https://www.bgs.ac.uk/datasets/biosphere-isotope-domains-gb/). As such, it is somewhat striking that this study makes no attempt at a more systematic, quantitative,

spatial approach to interpreting and presenting the isotopic provenance data. Such an approach would greatly improve the visualization, interpretation, and presentation of the isotope results by simply and effectively illustrating the areas of potential origin for each individual (or at least the British ones). Such an approach is not very complex and can be accomplished with a fairly simple application in ArcGIS, as demonstrated recently by a similar study combining skeletal isotope data and isoscapes (in a British context!) to trace the origins of individuals buried at Stonehenge (Snoeck et al. 2018).

We appreciate comments from all three reviewers about how best to present and interpret the provenancing data. The reviewers express very different opinions on this issue. We were torn on how best to approach the presentation of the data and how confidently to interpret. This remains a delicate balance to navigate as is demonstrated by the fact that some review comments stated that we need to be more ambitious and others that we need to be more cautious. Britain is indeed the most comprehensively mapped and we used the BGS multi-isotope querying tool to explore origins. On the basis of the three proxies this indicated that only one individual (FCS-09, with African ancestry) was consistent with British origins. This is, in practice, inconsistent with the data and demonstrates that we are not yet (generally, at least) in a position where we can query data to plot origins on a map. The manifold variables that affect these isotope proxies means we are in a situation of providing the most parsimonious explanations of origins and using ArcGIS approaches to plotting origins can over-simplify the complex process of exploring origins. We would like to show greater ambition in refining provenance and take a more solid, quantitative approach, but we are not sure the data can sustain it and it would go against comments of another reviewer. In addition, three of the authors have been involved in a study that has been criticised for overambitious refinement of origins (see Barclay and Brophy 2020, Archaeological Journal), so would rather err on the side of caution.

Based on the concerns detailed above, I recommend that the manuscript be accepted with minor revisions. I hope that the authors seriously consider the proposed suggestions for revision, and look forward to reading the revised and published version of this paper in Royal Society Open Science.

Reviewer #2
The manuscript by Scorrer and colleagues presents a study on the medieval warship Mary Rose. The manuscript attempts an isotope study in order to understand the origins of the crew to the ship. The article is a well written summary and the study scientifically sound, so that the whole manuscript gives a detailed insight into the crews diet and possible origins. However, there are some flaws in the study's design, since the team analysed carbon, nitrogen, sulphur, oxygen and strontium isotopes of eight individuals the sample numbers are very low and discrepancies in the data result in highly biased data. Therefore, there are no strong conclusions and the article is adding another layer of information without revealing origins or deeper understanding of the Tudor's warship. In addition the authors decided to include a fully unrelated craniometric ancestry estimation into the article, which fails to connect to the other results and is only another mosaic puzzle piece in the story, which does not fit with other results.

General remarks:
The data table 1 should be merged with table S1 since they both contain valuable information and only together these information are intuitive assessable. Additionally the table description needs to be more substantial.

We believe that combining table 1 and S1 will create too large a table so would like to keep these separate but agree with the reviewer that the data from both these tables should be better linked. We have signposted this in the table 1 caption (and have added more detail to the caption):

Table 1. Multi-isotope analysis results from dental samples of eight individuals from the Mary Rose (see electronic supplementary material, table S1 for contextual information on these individuals). Oxygen values were converted from carbonate ($\delta^{18}O_c$) to phosphate ($\delta^{18}O_p$) using the conversion equation set out in Chenery et al. [90].

The Figures need a higher resolution, Figures 1,3, and 4 need a higher resolution and better quality, my printouts were bad and even on screen were no distinctions between symbols.

For Figure 1 of the Mary Rose, unfortunately we only have permission from the Pepys Library to have the resolution of the image as 70 dpi. The quality of figures 3 and 4 have been improved for final submission.

The introduction to the Tudor kingdom and the mobility of the navy is excellent. It is well written, substantial yet not lengthy. The isotope background is alright, but lacks some introduction to isotope data from the studied period. There is a multitude of data published and a overview of available data would have been a good start for understanding the setting.

We have added the following to the end of section 1.4 to address this:

There has been little isotope work on post-medieval human remains in Britain (see [9, 53, 70, 71]). There is, however, a wealth of data for late medieval Britain, especially in terms of $\delta^{13}C$ and $\delta^{15}N$ [28, 29, 72-74], but also for $^{87}Sr/^{86}Sr$ and $\delta^{18}O$ [66, 75].

We have also clarified we are referring to the Tudor rather than medieval period when we say that direct evidence of human remains from this period is limited (at the end of section 1.3), but that we are happy to add any studies if we have missed some that relate to this period.

The study design is alright, given the importance and value of the samples, however, seven individuals are very few and therefore the robustness of such data will be limited. This is the major concern of the study, because the results will be only trustworthy, if there are no or limited numbers of outliers, but due to the nature of the historic background we would expect quite a number of outliers. This means there will be no background data for proper interpretation. Therefore, the general literature review of isotopic mobility studies is most

important.
The sample treatment and analytical details are highly detailed and should be cut to a minimum, additionally many of these methods have been published before and these should be acknowledged.

We appreciate that we have a long methods section as multiple methods were used. Other reviewers and the editor have asked for more methodological detail, so we are obliged to keep them in, though we agree that they disrupt the flow of the text.

The ancestry estimation by craniometric and morphoscopic methods should be excluded from the manuscript. Though valid to a certain point the sample number and variability in the skeletal remains are quite big and the interpretation results more in speculations than scientific conclusions. In my opinion the interpretation could be added in a side note, but not a full chapter.

In the absence of aDNA data (though ongoing research will add this in time), we deemed it important to explore ancestry to some degree though we accept that the interpretations are far from certain. However, the results for FCS-09 are convincing and this does add another biographical element to the crew. Both other reviewers praised the ancestry estimation and the value it added. As a result we would like to retain this element. Furthermore, the results of this analysis were included in the museum interpretation and a Channel 4 documentary on the Mary Rose, and so we feel that the methods used should be peer-reviewed and published so others can evaluate interpretations across the data.

The conclusion are just a summary and are relativizing the own data. In my opinion the results are only limited and this needs to be addressed. Additionally the authors have started a data set for the Mary Rose material which needs to be expanded in put into the larger context.

We are grateful for this observation and have made major changes to the conclusion. It is now less of a summary and better emphasises that our sample is small and that more work on the Mary Rose collection needs to be done. We hope that this addresses the reviewer's comment.

Minor comments:
The collagen was not ultrafiltered, this is usually sufficient for carbon and nitrogen isotope results, however, for sulphur isotopic results this could be problematic. Additionally the salt water could have compromised the materials and therefore an additional ultrafiltration step seems wise. My concern is related to the correlation of sulphur isotope values with sulphur content in collagen. The highest sulphur isotope values also revealed the highest sulphur contents. This could be indicative for seawater sulphate intrusion, therefore these data should be questioned and double checked.

This is an interesting point. We are unaware of evidence that ultrafiltration is necessary for sulphur isotope analysis of teeth deposited in marine environments. All quality control criteria were met, as demonstrated in the paper. We are very confident that our results are valid. The range of sulphur values would be difficult to explain if diagenesis had

occurred and therefore we are confident that they are biogenic. In addition, diagenetic alteration would skew C:S and N:S ratios.

The strontium results are nice, but the interpretation and presentation lacks ambition. I would recommend to use literature data for comparison and additional arguments. In my opinion these data have been neglected in the interpretation. Similarly the oxygen isotope data, which are more problematic, but in itself have some value, which needs to be addressed.

We appreciate comments from all three reviewers about how best to present and interpret the provenancing data. The reviewers express very different opinions on this issue. We were torn on how best to approach the presentation of the data and how confidently to interpret. This remains a delicate balance to navigate as is demonstrated by the fact that some review comments stated that we need to be more ambitious and others that we need to be more cautious. Britain is indeed the most comprehensively mapped and we used the BGS multi-isotope querying tool to explore origins. On the basis of the three proxies this indicated that only one individual (FCS-09, with African ancestry) was consistent with British origins. This is, in practice, inconsistent with the data and demonstrates that we are not yet (generally, at least) in a position where we can query data to plot origins on a map. The manifold variables that affect these isotope proxies means we are in a situation of providing the most parsimonious explanations of origins and using ArcGIS approaches to plotting origins can over-simplify the complex process of exploring origins. We would like to show greater ambition in refining provenance and take a more solid, quantitative approach, but we are not sure the data can sustain it and it would go against comments of another reviewer. In addition, three of the authors have been involved in a study that has been criticised for overambitious refinement of origins (see Barclay and Brophy 2020, Archaeological Journal), so would rather err on the side of caution.

The suggested changes are not to major in my opinion and therefore should be addressed and the manuscript altered accordingly. After doing so, in my regards to the high quality of the manuscript's style there is no issue with publication.

Reviewer #3

Scorrer et al. have prepared a well-written manuscript that succinctly presents and very adequately interprets multi-isotope and morphometric skeletal data from a relatively small (but well selected) sample of humans from the Mary Rose wreck. This paper is an important contribution to the growing literature involving these datasets, and more specifically helps to add additional scientific value to our understanding about the life-ways (i.e. origins) of individuals from the Tudor time period. I have added specific comments/corrections to the attached .pdf of the manuscript, and mention a few of these items here.

There are no major concerns with the publication of this manuscript providing these minor corrections made and/or considered to help substantiate the interpretation of the isotope data. The authors have been particularly thorough in their realistic evaluation of the morphometric determination using the existing software and databases available for this

purpose (e.g. Fordisc), which are not specific to archaeological populations. The additional information on this part of the study contained in the supplemental was much appreciated and necessary.

Finally, the balance between offering specific (likely) geographic origins for each of the individuals sampled with a recognition of equifinality inherent in individual, or combined, isotope systems (δ13C, δ15N, δ18O, δ34S, and 87Sr/86Sr) is reasonably done. I have suggested statistical treatment of the isotope data should be attempted/worked through via appropriate non-parametric methods to help further support the author's suggestion of origins within or outside 'Britain'. Given the additional information available on each of the individuals that are part of this study, one could consider a possible bayesian approach to determining 'local' versus 'non-local' in this context.

We are grateful to the reviewer for highlighting the potential. We consulted a statistician at Cardiff University and they advised against tests of difference on such a small sample. The power of the dataset is too poor and it is certainly not standard practice to conduct tests of difference on such small samples. We have added the word 'deemed' to section 3.2 to show that this was our decision.

Overall I enjoyed the paper and how the authors have approached this research, and I look forward to seeing it published in its revised format.

We have made all the minor revisions suggested by reviewer 3 throughout the .pdf of the manuscript. Below are responses to comments which require more detailed replies.

Section 2.3 comments:

Reviewer 3: Just a method point here. Not sure why this particular approach was taken to remove additional Ca from the samples. One could simply include additional washes of 8M HNO3 through the columns (after the sample was loaded) to remove Ca.

This is an interesting methodological point. From testing and general experience with Eichrom/Triskem organic bead resins, the effectivity of removal of complex matrix does not follow the Kd curves exactly. Particularly for removing major matrix, as in this case Ca, there is a saturation of Ca complexing and adding more 8N HNO3 will not remove the remaining Ca. However, by drying samples down and reloading on new resin the remaining Ca can be nearly completely eluted. Sentence has been changed to:

Samples were placed on a hotplate (120°C) to dry overnight before this process was repeated for a second pass for the effective removal of any remaining traces of Ca.

Reviewer 3: Presumably SRM 1400 was also prepared in the same way as the enamel samples. If so, please state this. I would suggest that both SRM 987 and 1400 are being used

for accuracy checks here since neither has certified values and previously published values are being reported to help with data quality assurance.

Apologies – we should have clarified this. Yes, 1400 was processed through chemistry in the same way, This has now been made clear in the text. The measured SRM 987 as the primary standard cannot really be used as a test of accuracy but we agree with the reviewer that it may appear incorrect to say we use the 1400 for accuracy check. What we actually are doing is to check the accuracy (as to within published values) and robustness of our sample processing and normalisation to the measured NBS987 primary standard. We have now changed the sentence to reflect this:

Accuracy of the NIST SRM 987 normalisation and the chemistry processing was assessed by repeat measurements of $^{87}Sr/^{86}Sr$ ratio in NIST SRM 1400 (Bone Ash, processed through chemistry similar to the unknown samples), giving an average $^{87}Sr/^{86}Sr$ ratio of 0.713111 ±0.000014 ($2\sigma_N$, n=5), which is consistent with the published value (0.713126±0.000017 [80]).

Reviewer 3: No pretreatment of the enamel was carried out prior to acid digestion on the Kiel device? Typically, some form of pretreatement is carried out when analysing carbonate in teeth. If this is argued to not be necessary, then appropriate studies/literature should be included here to indicate this.

This is another interesting methodological point and there has been much debate about carbonate pre-treatments. We opted for no pre-treatment to remain in line with Chenery et al 2012 (ref 83), they cleaned their material with DI only. In addition, changes to isotope composition due to pretreatment can be large and variable (Pellegrini and Snoek 2016). In our view we could reduce potentially altering affects by retaining samples untreated material. Pellegrini and Snoek (2016) is now cited in the paper and we mention the potential altering effects of pre-treatment.

Reviewer 3: Were the errors associated with each step of this process (d18O measurement, conversion from VPDB to VSMOW, and then from d18Ocarb to d18Ophos) propagated and reported?

Thank you for highlighting this omission. We have added more information about associated error, including the standard deviation of replicate measurements and the error related to Chenery's regression conversion:

The standard deviation of replicate δ18O measurements is 0.049‰. The δ18Ocarbonate values were converted to δ18Ophosphate (δ18Op) values (following [89]) to allow for comparison with other datasets. The error on the calculated δ18Op values is 0.24‰, based on the analytical error and the error in the conversion regression equation [89].

We have also added extended oxygen data in the supplementary material.

Section 2.4 comments:

Reviewer 3: Given the vagaries associated with %N and %C determinations on an EA, I would expect a calibration of materials with different % level contents (i.e. that span the range of values expected in collagen at the very least) to be used in these determinations.

To address this, we have changed the sentence to:

The 1σ (n=54) reproducibility was ±0.06 for δ13C and ±0.07 for δ15N. Different weights of caffeine were analysed to establish a calibration equation for the abundance of C and N against signal intensity in the mass spectrometer, which was used to calculate %N and %C in actual samples. The content of caffeine (28.85%N, 49.48%C) was calculated from its chemical formula (C8H10N4O2).

Reviewer 3: While this comment doesn't require the authors to re-do their analyses, I would suggest for future isotope related analyses they incorporate both calibration and check standards into their analytical d13C, d15N, and concentration determinations. Additionally, calculation of the total uncertainty in their measurement can follow the recommendations and procedure in Szpak et al. (2017) "Best practices for calibrating and reporting stable isotope measurements in archaeology." JAS:R 13:609-616.

Thank you for this comment. We have made the additions above and believe that this essentially follows what the Szpak paper recommends.

Section 3.1 comment:

Reviewer 3: The mean-median difference of 0.3 per mil is really not that significant when the errors associated with measurement of d18Ocarb, and the value conversions from VPDB to VSMOW scales, and then to d18Op are factored in.

We agree that this is not a marked difference. However, any errors are not associated with the conversion from VPDB to VSMOW, as it's purely a numerical conversion between two different scales; the errors are related to measurement of $^{18}O_c$ and the conversion to $^{18}O_p$ and these are reported in the cited literature.

Section 3.2 comments:

Reviewer 3: How were 'outliers' determined? Was this through simiply looking at the data, or where statistical tests or evaluations of the dataset carried out?

These outliers were identified through basic observation, as statistical testing was considered inappropriate for such a small dataset.

Reviewer 3: There are appropriate statistical tests that can be performed on small datasets such as these, accounting for non-normal distributions and using post-hoc analyses to mitigate the smaller sample size and distribution. Some examples include Wilcoxon rank and Kruskal-Wallis, for starters.

We are grateful to the reviewer for highlighting the potential. We consulted a statistician at Cardiff University and they advised against test of difference on such a small sample. The power of the dataset is too poor and it is certainly not standard practice to conduct tests of difference on such small samples. We have added the word 'deemed' to section 3.2 to show that this was our decision.

Section 4 comment:

Reviewer 3: I'm sure the authors are aware high d34S values (>14 per mil) would also indicated an origin consistent with living on limestone, which occurs throughout the UK. As such, d34S becomes less useful for determining origins in this particular case.

We are aware of some evidence for a relationship between limestone lithology and sulphur isotope values. However, the data are fairly weak and inconsistent and therefore we do not think they can be relied on yet. Evans et al. (2018). do not map limestone regions as 14+. Zazzo et al. (2011) did not identify this pattern in their study of modern sheep and nor is it mentioned in Nehlich's (2015) review of sulphur isotope research. Guiry and Szpak (2020) and Zazzo et al. (2011) have demonstrated that seaspray (and thus coastal proximity) is the dominant variable in driving high sulphur values. Madgwick et al. 2019 (57) measure S in 6 plant samples from limestone areas and all have values between 6 and 9 (in line with the Evans et al. 2018 map for inland limestone zones). In short, the evidence for high values on limestone is limited.

Section 4.1 comment:

Reviewer 3: Since the baseline areas overlap in d18Op values, the authors should be more cautious in their interpretation of these data to fall within any specific geographic region in the UK. I would also recommend the authors make some attempt to determine whether or not the overlap (or lack there of) between these samples and the areas indicated (east versus west of Britain) actual have some statistical significance; perhaps even using estimate plots of the median (and distributions of the data) would be helpful here.

The text has been changed to be less firm in interpretation and caveat has been added again. Given the small sample size, we are reluctant to statistically test our data and rely on Evans et al. (2012) and Pellegrini et al. (2016) who both highlight that east and west Britain can be broadly differentiated. We acknowledge this difference is only sufficient for us to state that it is 'likely' that these individuals are from the west/south.
